# Psychological Distress Among US-Born and Non–US-Born Black or African American Adults in the US

**DOI:** 10.1001/jamanetworkopen.2025.6558

**Published:** 2025-04-28

**Authors:** Maryam Elhabashy, David Adzrago, Faustine Williams

**Affiliations:** 1 Division of Intramural Research, National Institute on Minority Health and Health Disparities, Bethesda, Maryland

## Abstract

**Importance:**

Limited research explores within-group and between-group differences in the prevalence of and factors associated with psychological distress among Black or African American adults, especially by nativity.

**Objective:**

To estimate the prevalence of moderate-to-severe (hereafter, moderate-severe) psychological distress and to assess factors associated with increased risk among Black or African American adults according to nativity.

**Design, Setting, and Participants:**

This cross-sectional study drew data from the 2005 to 2018 National Health Interview Surveys. The study analyzed national household probability samples of the civilian noninstitutionalized Black or African American adult population aged 18 years or older, including US-born and non–US-born subgroups, between January to December 2005 and January to December 2018. Data analysis was performed from November 2023 to January 2025.

**Exposures:**

Birthplace (ie, US-born if born in the US; non–US-born if born outside the US, including US territories). Risk factors included sociodemographic, socioeconomic, and health behavior factors.

**Main Outcomes and Measures:**

The primary outcome was moderate-severe psychological distress status based on self-reported responses to the Kessler Psychological Distress Scale. Odds ratios (ORs) with 95% CIs were reported as estimates to determine the computed associations across logistic regression models.

**Results:**

A total of 49 820 individuals (43 885 born in the US and 5935 born outside the US) were analyzed. Overall, 21.9% of the sample (11 079 individuals) experienced moderate-severe psychological distress, with a higher prevalence among US-born (10 037 individuals [22.6%]) than non–US-born (1042 individuals [17.4%]) individuals. Individuals aged 65 years or older (especially US-born; OR, 0.51; 95% CI, 0.44-0.58) and male individuals (especially non–US-born; OR, 0.68; 95% CI, 0.56-0.82) had lower odds of experiencing moderate-severe psychological distress. Unemployment (OR, 1.91; 95% CI, 1.80-2.03) and having less than a college education were associated with higher odds of moderate-severe psychological distress across the subgroups, especially among US-born individuals. Current and former smoking was associated with higher odds of moderate-severe psychological distress, with greater odds among non–US-born individuals than among US-born and overall Black or African American individuals. Current and former alcohol drinking was associated with higher odds among only the general population (current drinking, OR, 1.37 [95% CI, 1.29-1.47]; former drinking, OR, 1.26 [95% CI, 1.16-1.37]) and US-born individuals (current drinking, OR, 1.45 [95% CI, 1.36-1.56]; former drinking, OR, 1.29 [95% CI, 1.19-1.41]), with higher ORs among US-born population.

**Conclusions and Relevance:**

In this cross-sectional study of differences in moderate-severe psychological distress by nativity among Black or African American adults, more pronounced risks were observed among US-born individuals. Longitudinal studies and data disaggregation could further elucidate health differences to improve cultural competence and adaptability in mental health research and interventions.

## Key Points

**Question:**

Do the prevalence of and factors associated with moderate-to-severe psychological distress differ between US-born and non–US-born Black or African American individuals?

**Findings:**

In this cross-sectional study that included 49 820 Black or African American adults, a higher proportion of US-born individuals experienced moderate-severe psychological distress than non–US-born individuals. Notable characteristics associated with higher risks among US-born individuals included younger age, divorce or separation, unemployment, lower educational level, lack of health insurance, and drinking alcohol.

**Meaning:**

These findings suggest that interventions targeting mental health among Black or African American adults may benefit from considering between-group and within-group differences based on nativity.

## Teaser

This cross-sectional study estimates the prevalence of moderate-to-severe psychological distress and factors associated with its risk among Black or African American adults according to nativity.

## Introduction

Psychological distress is a condition that constitutes symptoms of stress, anxiety, and depression, which often co-occur.^1,2,3^ Previous studies^4,5,6^ have established that Black or African American individuals have disproportionately higher burdens of mental health disorders than their White US counterparts. However, there is limited literature on nativity-based disparities in mental health conditions like psychological distress among Black or African American individuals, despite well-established knowledge of the extent and impact of health disparities in this population.^7,8^

There is a complex association between race and psychological distress. Although some studies^5,9,10,11^ have found that Black or African American individuals have lower prevalence and risk of psychological distress than their White counterparts, others^12,13^ have reported higher psychological distress among Black or African American individuals. Black or African American individuals have often been examined as a monolithic group, potentially neglecting the heterogeneity in findings and populations.^14,15,16^ In 2022, 11.8% of Black or African American individuals were non–US-born, and the proportion is projected to be 13.0% in 2035 and 16.6% in 2060.^17^ This necessitates more research that considers the diversity in this population, which can have important implications for mental health disparities in the increasingly diverse US population.

Although collecting and analyzing disaggregated data remains less common in public health research,^18^ it can be especially important for identifying and addressing the needs of at-risk populations. The current study’s objectives were to (1) estimate the prevalence of psychological distress among Black or African American adults according to their nativity and (2) assess the associations of psychological distress with sociodemographic, socioeconomic, and health behavior factors across the general Black or African American population and stratified by nativity. This study aims to contribute to the understandings of Black or African American experiences of psychological distress and mental health disparities.

## Methods

### Data Sources and Sample

**Figure 1.**
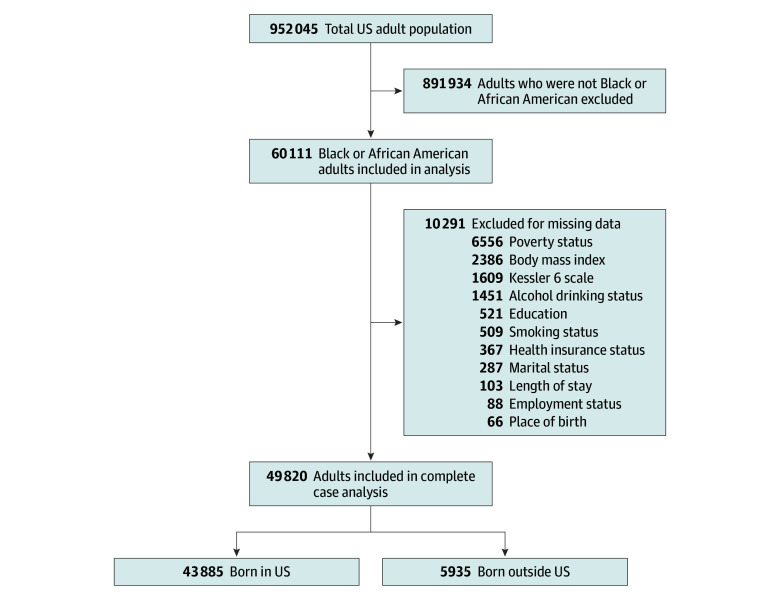
Participant Enrollment Flowchart

In this cross-sectional study, we analyzed data from the 2005 to 2018 National Health Interview Survey (NHIS), which uses stratified, complex clustered sampling to conduct a nationally representative survey among US noninstitutionalized civilians residing within the 50 states, US territories, and the District of Columbia.^19,20,21^ Data collected beyond 2018 were not used in this study because of content and structure changes that rendered them incompatible with earlier NHIS data. The pooled data included a sample of 952 045 adults, with 60 111 individuals self-identifying as Black or African American adults. Individuals with missing responses for any variables of interest were excluded (Figure 1).^22,23,24,25^ The inclusion of US territories in the non–US-born category is based on evidence that governance and health care systems in the territories differ substantially from those in the US states.^26,27,28^ This study followed the Strengthening the Reporting of Observational Studies in Epidemiology (STROBE) reporting guidelines.^29^ The study did not require individual-level consent or institutional review board approval, because it used publicly available, deidentified, anonymous secondary data, in accordance with 45 CFR §46.^30^

### Moderate-to-Severe Psychological Distress

Moderate-to-severe (hereafter, moderate-severe) psychological distress was measured using the Kessler Psychological Distress Scale, which is a 6-item scale that uses a 5-point Likert-type rating ranging from 0 (none of the time) to 4 (all of the time). The 6 items involve how often respondents felt sad, nervous, restless, hopeless, worthless, or that everything was an effort in the past 30 days.^31^ Total scores range from 0 to 24, with higher scores indicating greater psychological distress.^31,32,33^ Scores less than 5 indicate no to mild psychological distress, and scores of 5 or higher indicate moderate-severe psychological distress.^31,32,33^

### Factors Associated With Moderate-Severe Psychological Distress

This study assessed 12 factors associated with moderate-severe psychological distress. Sociodemographic factors included age (18-25, 26-34, 35-44, 45-54, 55-64, or ≥65 years), sex (male or female), marital status (single or never married, married or living with a partner, separated, widowed, or divorced), and region of residence (Northeast, North Central or Midwest, South, and West). Socioeconomic factors included employment status (employed or unemployed), poverty status (income below poverty threshold or at or above poverty threshold), and education (less than high school, high school graduate, some college or associate degree, or college degree or higher). Health behaviors and related factors included health insurance status (insured or uninsured), body mass index (BMI; calculated as weight in kilograms divided by height in meters squared; underweight or normal, <25; overweight, ≥25 and <30; or obesity, ≥30), physical activity (physically active vs inactive or insufficiently active), alcohol drinking status (current [had 1-11 or more drinks in the past year], former [had at least 12 drinks in any year in lifetime, but none in past year], or never [had fewer than 12 drinks in lifetime]), and cigarette smoking status (current [ever smoked 100 cigarettes in lifetime and currently], former [ever smoked 100 cigarettes in lifetime but not currently], or never [never smoked 100 cigarettes in lifetime]).

Physical activity was assessed according to the 2018 Physical Activity Guidelines for Americans’ moderate to vigorous leisure-time physical activity requirements.^34^ Participants were categorized as active (met requirements) or insufficiently active or inactive (did not meet requirements).

### Statistical Analysis

Data analysis was performed from November 2023 to January 2025. Sampling weights were applied with the strata and primary sampling unit to account for the complex survey sampling design and nonresponses, ensuring statistically accurate estimates, including SEs, percentages, and other effect estimates.^21^ Only a sample of the US population was selected to complete the survey, some of whom did not respond or complete the survey, resulting in nonresponses. To address nonresponse, each participant was assigned a weight reflecting the number of individuals they represented in the target population. The overall sampling weights (ie, the sum of each participant’s weight) accounted for the general target population.^21^ Unweighted frequencies and their corresponding weighted percentages were computed using descriptive and bivariate statistics before testing between-group differences in prevalence using Rao-Scott χ^2^ tests.^35,36^ We used logistic regression models to examine the associations between psychological distress and its related factors among the overall sample of the Black or African American population, as well as the non–US-born and US-born subgroups, respectively. We also examined the interaction between nativity and each factor, adjusting for the remaining factors. Odds ratios (ORs) with 95% CIs were reported as estimates to determine the computed associations across the logistic regression models,^37^ with statistical significance at 2-tailed *P* < .05. All statistical analyses were conducted using STATA statistical software version 18.0 (StataCorp) with the commands svy (to incorporate the strata and primary sampling unit) and subpop (eg, for weighted and subpopulation analyses).

## Results

### Population Characteristics

There were 49 820 complete cases of US Black or African American adult respondents who answered questions regarding psychological distress and related factors through the NHIS 2005 to 2018; 43 885 were born in the US, and 5935 were born outside the US (Table 1). The overall sample was majority female (29 780 individuals [58.1%]), employed (28 035 individuals [58.9%]), physically inactive or insufficiently active (48 883 individuals [98.0%]), current alcohol drinkers (27 211 individuals [56.4%]), and never smokers (31 881 individuals [65.7%]). They mostly lived in the Southern region of the US (29 473 individuals [58.3%]), had incomes at or above the poverty threshold (36 046 individuals [73.3%]), had health insurance (41 268 individuals [83.0%]), and had some college or an associate’s degree (16 379 individuals [33.6%]).

**Table 1.** Psychological Distress Prevalence by Sociodemographic, Socioeconomic, and Behavioral Characteristics of Non–US-Born and US-Born Black or African American Adult Population

Characteristic	Total (N = 49 820)			Non–US-born (n = 5935)			US-born (n = 43 885)		
	Participants, No. (%) [95% CI]^a^		*P* value	Participants, No. (%) [95% CI]^a^		*P* value	Participants, No. (%) [95% CI]^a^		*P* value
	Overall sample	Psychological distress (n = 11 079)		Overall sample	Psychological distress (n = 1042)		Overall sample	Psychological distress (n = 10 037)	
Psychological distress									
Yes	11 079 (21.9) [21.4-22.5]	NA	NA	NA	NA	NA	NA	NA	NA
No	38 741 (78.1) [77.5-78.6]	NA		NA	NA		NA	NA	
Nativity									
Non–US-born	5935 (11.9) [11.3-12.5]	1042 (17.4) [16.2-18.7]	<.001	NA	NA	NA	NA	NA	NA
US-born	43 885 (88.1) [87.5-88.7]	10 037 (22.6) [22.0-23.1]		NA	NA		NA	NA	
Age, y									
18-25	6187 (14.2) [13.6-14.9]	1407 (22.6) [21.4-23.9]	<.001	580 (11.2) [10.1-12.4]	111 (19.8) [16.3-23.9]	.65	5607 (14.6) [13.9-15.4]	1296 (22.9) [21.6-24.3]	<.001
26-34	8598 (18.9) [18.4-19.4]	1957 (22.5) [21.5-23.6]		1205 (21.1) [19.9-22.3]	213 (17.0) [14.7-19.6]		7393 (18.6) [18.1-19.2]	1744 (23.4) [22.2-24.6]	
35-44	8796 (18.0) [17.5-18.4]	1866 (21.1) [20.1-22.1]		1420 (23.4) [22.2-24.6]	249 (18.0) [15.7-20.6]		7376 (17.2) [16.8-17.7]	1617 (21.7) [20.6-22.8]	
45-54	9159 (17.8) [17.4-18.2]	2282 (24.1) [23.1-25.2]		1232 (20.0) [18.9-21.2]	210 (16.6) [14.2-19.4]		7927 (17.5) [17.1-17.9]	2072 (25.3) [24.2-26.4]	
55-64	8061 (15.1) [14.8-15.5]	1992 (24.0) [22.9-25.2]		752 (12.4) [11.4-13.5]	129 (16.3) [13.7-19.2]		7309 (15.5) [15.1-15.9]	1863 (24.8) [23.6-26.1]	
≥65	9019 (16.0) [15.4-16.5]	1575 (17.2) [16.2-18.2]		746 (12.0) [11.0-13.0]	130 (17.4) [14.3-20.9]		8273 (16.5) [15.9-17.1]	1445 (17.2) [16.2-18.2]	
Sex									
Male	20 040 (41.9) [41.3-42.5]	3821 (18.6) [17.9-19.3]	<.001	2732 (48.2) [46.8-49.7]	403 (14.0) [12.5-15.6]	<.001	17 308 (41.0) [40.4-41.7]	3418 (19.4) [18.6-20.2]	<.001
Female	29 780 (58.1) [57.5-58.7]	7258 (24.3) [23.7-25.0]		3203 (51.8) [50.3-53.2]	639 (20.6) [18.8-22.5]		26 577 (59.0) [58.3-59.6]	6619 (24.8) [24.1-25.5]	
Marital status									
Divorced	8068 (15.4) [15.1-15.8]	1935 (23.6) [22.6-24.7]	<.001	825 (14.2) [13.2-15.2]	176 (21.5) [18.0-25.4]	<.001	7243 (15.6) [15.2-16.0]	1759 (23.9) [22.8-25.0]	<.001
Widowed	4623 (8.3) [7.9-8.7]	1000 (21.4) [19.9-23.0]		290 (4.4) [3.8-5.0]	64 (23.4) [18.3-29.4]		4333 (8.9) [8.4-9.3]	936 (21.3) [19.7-22.9]	
Separated	2887 (5.5) [5.3-5.8]	842 (28.2) [26.4-30.1]		428 (6.9) [6.2-7.7]	101 (20.8) [17.0-25.2]		2459 (5.4) [5.1-5.6]	741 (29.5) [27.5-31.6]	
Married or living with partner	15 739 (32.1) [31.4-32.8]	2855 (17.8) [17.1-18.5]		2765 (47.1) [45.5-48.6]	374 (13.1) [11.7-14.7]		12 974 (30.0) [29.3-30.7]	2481 (18.8) [18.0-19.6]	
Single or never married	18 503 (38.6) [37.9-39.4]	4447 (23.9) [23.2-24.7]		1627 (27.5) [26.1-28.9]	327 (20.9) [18.7-23.3]		16 876 (40.1) [39.3-40.9]	4120 (24.2) [23.4-25.0]	
Region of residence									
Northeast	7327 (15.3) [14.5-16.2]	1520 (20.7) [19.5-21.9]	.005	2138 (36.1) [33.7-38.5]	390 (18.4) [16.1-21.0]	.26	5189 (12.5) [11.7-13.4]	1130 (21.6) [20.2-23.0]	.02
North Central or Midwest	8365 (18.0) [17.0-19.1]	1966 (23.6) [22.4-24.8]		665 (11.7) [10.2-13.3]	123 (17.2) [14.1-20.8]		7700 (18.9) [17.8-20.0]	1843 (24.1) [22.8-25.4]	
South	29 473 (58.3) [56.8-59.7]	6530 (21.7) [21.0-22.3]		2424 (41.7) [39.1-44.3]	398 (16.1) [14.5-17.8]		27 049 (60.5) [59.0-62.1]	6132 (22.2) [21.5-22.9]	
West	4655 (8.4) [7.9-8.9]	1063 (22.7) [21.1-24.5]		708 (10.6) [9.4-11.9]	131 (19.7) [15.7-24.4]		3947 (8.1) [7.6-8.6]	932 (23.3) [21.5-25.2]	
Employment status									
Employed	28 035 (58.9) [58.1-59.6]	4757 (17.0) [16.5-17.6]	<.001	4103 (70.0) [68.7-71.4]	618 (15.0) [13.7-16.3]	<.001	23 932 (57.4) [56.6-58.2]	4139 (17.4) [16.8-18.0]	<.001
Not employed	21 785 (41.1) [40.4-41.9]	6322 (29.0) [28.1-29.8]		1832 (30.0) [28.6-31.3]	424 (23.1) [20.6-25.8]		19 953 (42.6) [41.8-43.4]	5898 (29.5) [28.7-30.4]	
Health insurance status									
Insured	41 268 (83.0) [82.5-83.5]	8795 (21.0) [20.5-21.6]	<.001	4547 (77.9) [76.5-79.4]	779 (16.8) [15.6-18.2]	.06	36 721 (83.7) [83.2-84.1]	8016 (21.6) [21.0-22.1]	<.001
Uninsured	8552 (17.0) [16.5-17.5]	2284 (26.4) [25.3-27.5]		1388 (22.1) [20.6-23.5]	263 (19.5) [16.9-22.4]		7164 (16.3) [15.9-16.8]	2021 (27.6) [26.5-28.9]	
Education									
Less than high school	9625 (17.7) [17.1-18.4]	2881 (29.6) [28.5-30.7]	<.001	1128 (16.6) [15.5-17.9]	251 (21.3) [18.5-24.4]	<.001	8497 (17.9) [17.2-18.6]	2630 (30.6) [29.4-31.8]	<.001
High school graduate	14 424 (28.3) [27.8-28.9]	3387 (23.2) [22.4-24.1]		1439 (23.6) [22.3-25.0]	278 (19.2) [16.8-21.8]		12 985 (29.0) [28.4-29.6]	3109 (23.7) [22.8-24.6]	
Some college or associate’s degree	16 379 (33.6) [32.9-34.2]	3585 (21.9) [21.1-22.8]		1747 (30.0) [28.6-31.4]	310 (18.6) [16.6-20.8]		14 632 (34.1) [33.4-34.8]	3275 (22.3) [21.4-23.2]	
College degree or higher	9392 (20.3) [19.7-21.0]	1226 (13.5) [12.7-14.4]		1621 (29.8) [28.3-31.3]	203 (12.6) [10.8-14.7]		7771 (19.1) [18.4-19.7]	1023 (13.7) [12.8-14.7]	
Poverty status									
Income less than poverty threshold	13 774 (26.7) [26.0-27.5]	4611 (33.1) [32.1-34.1]	<.001	1412 (22.6) [21.2-24.1]	356 (25.3) [22.6-28.3]	<.001	12 362 (27.3) [26.5-28.1]	4255 (33.9) [32.9-35.0]	<.001
Income greater than or equal to poverty threshold	36 046 (73.3) [72.5-74.0]	6468 (17.9) [17.4-18.4]		4523 (77.4) [75.9-78.8]	686 (15.1) [13.9-16.4]		31 523 (72.7) [71.9-73.5]	5782 (18.3) [17.8-18.8]	
Body mass index^b^									
Underweight or normal (<25)	13 686 (27.9) [27.4-28.4]	2968 (21.3) [20.5-22.2]	<.001	2051 (34.8) [33.4-36.2]	330 (16.0) [14.2-18.0]	<.001	11 635 (27.0) [26.5-27.5]	2638 (22.2) [21.3-23.2]	<.001
Overweight (≥25 and <30)	16 551 (33.2) [32.7-33.7]	3218 (19.2) [18.5-20.0]		2399 (40.6) [39.2-42.0]	380 (15.2) [13.5-17.1]		14 152 (32.2) [31.7-32.7]	2838 (19.9) [19.1-20.7]	
Obesity (≥30)	19 583 (38.9) [38.3-39.4]	4893 (24.7) [24.0-25.4]		1485 (24.7) [23.4-26.0]	332 (23.1) [20.5-25.8]		18 098 (40.8) [40.2-41.4]	4561 (24.9) [24.1-25.6]	
Physical activity									
Inactive or insufficient	48 883 (98.0) [97.8-98.1]	10 863 (21.9) [21.4-22.4]	.85	5826 (97.9) [97.4-98.3]	1021 (17.4) [16.1-18.7]	.81	43 057 (98.0 97.8-98.1]	9842 (22.5) [22.0-23.1]	.90
Physically active	937 (2.0) [1.9-2.2]	216 (22.2) [19.4-25.3]		109 (2.1) [1.7-2.6]	21 (18.4) [11.8-27.4]		828 (2.0) [1.9-2.2]	195 (22.8) [19.7-26.2]	
Alcohol drinking status									
Never	14 179 (27.7) [27.1-28.2]	2711 (19.0) [18.1-19.8]	<.001	2425 (39.8) [38.1-41.5]	427 (18.2) [16.4-20.1]	.005	11 754 (26.0) [25.4-26.6]	2284 (19.1) [18.2-20.1]	<.001
Former	8430 (16.0) [15.6-16.4]	2017 (23.2) [22.2-24.3]		634 (10.3) [9.5-11.3]	132 (21.8) [18.4-25.7]		7796 (16.8) [16.3-17.2]	1885 (23.4) [22.3-24.4]	
Current	27 211 (56.4) [55.7-57.1]	6351 (23.0) [22.4-23.7]		2876 (49.9) [48.2-51.5]	483 (15.9) [14.3-17.7]		24 335 (57.2) [56.5-58.0]	5868 (23.9) [23.2-24.5]	
Smoking status									
Never	31 881 (65.7) [65.0-66.4]	5994 (18.7) [18.1-19.3]	<.001	4855 (82.2) [81.1-83.3]	793 (16.2) [15.0-17.6]	<.001	27 026 (63.5) [62.8-64.2]	5201 (19.1) [18.5-19.7]	<.001
Former	7849 (14.8) [14.4-15.3]	1716 (21.7) [20.6-22.7]		588 (9.7) [8.8-10.6]	116 (19.9) [16.3-24.2]		7261 (15.5) [15.1-16.0]	1600 (21.8) [20.7-22.9]	
Current	10 090 (19.5) [18.9-20.0]	3369 (33.1) [32.0-34.2]		492 (8.1) [7.3-9.0]	133 (26.4) [22.1-31.1]		9598 (21.0) [20.4-21.6]	3236 (33.4) [32.3-34.6]	

Abbreviation: NA, not applicable.

^a^ Frequencies are unweighted, and percentages are weighted.

^b^ Body mass index is calculated as weight in kilograms divided by height in meters squared.

Subgroup differences in the population were observed for age, marital status, and BMI. The US-born subgroup was mostly aged 26 to 34 years (7393 individuals [18.6%]) and single or never married (16 876 individuals [40.1%]). The non–US-born subgroup was mostly aged 35 to 44 years (1420 individuals [23.4%]) and married or living with a partner (2765 individuals [47.1%]). For BMI, 40.8% of the US-born participants had obesity (18 098 individuals), and 40.6% of non–US-born participants had overweight (2399 individuals).

### Prevalence of Moderate-Severe Psychological Distress

Overall, 21.9% of the sample (11 079 individuals) experienced moderate-severe psychological distress. The US-born subgroup (10 037 individuals [22.6%]) reported a higher prevalence of moderate-severe psychological distress compared with the non–US-born subgroup (1042 individuals [17.4%]) (Table 1). Across the overall sample and subgroups, significantly higher prevalences of psychological distress were observed among individuals who were female, were unemployed, had less than high school education, had incomes below the poverty threshold, had obesity, or were current smokers. Among the overall population and the US-born group, higher prevalence was observed among current alcohol drinkers (5868 individuals [23.9%]), separated individuals (741 individuals [29.5%]), individuals who were aged 45 to 54 years (2072 individuals [25.3%]), uninsured individuals (2021 individuals [27.6%]), and those living in the North Central or Midwestern region (1843 individuals [24.1%]). Among non–US-born individuals, higher prevalence was observed among former alcohol drinkers (132 individuals [21.8%]) and widowed individuals (64 individuals [23.4%]). Among non–US-born individuals, there were no statistically significant differences by age, region of residence, or health insurance status. Furthermore, there were no statistically significant differences in prevalence based on physical activity across all groups.

### Factors Associated With Moderate-Severe Psychological Distress

There was a significant association between nativity and psychological distress, with non–US-born individuals, irrespective of their length of stay in the US, having lower odds of moderate-severe psychological distress compared with US-born individuals (eTables 1 and 2 in Supplement 1). Physical activity was the only variable not significantly associated with psychological distress (Table 2). In the adjusted models, similar patterns of associations were observed across all 3 groups: the overall population, non–US-born, and US-born (Table 2). Within the overall sample, lower odds of psychological distress were observed among individuals who were male (OR, 0.68; 95% CI, 0.56-0.82) vs female, had incomes at or above the poverty threshold (OR, 0.67; 95% CI, 0.64-0.72) vs below poverty threshold, were aged 65 years or older (OR, 0.51; 95% CI, 0.44-0.58) vs aged 18 to 25 years, were insured (OR, 0.91; 95% CI, 0.85-0.98) vs uninsured, and were married or living with a partner (OR, 0.91; 95% CI, 0.85-0.97) vs single or never married. In contrast, higher odds of psychological distress were found for individuals who were unemployed (OR, 1.91; 95% CI, 1.80-2.03) vs employed, had obesity (OR, 1.24; 95% CI, 1.16-1.32) vs those who were underweight or had normal weight, were aged 26 to 34 years (OR, 1.10; 95% CI, 1.01-1.21) or 45 to 54 years (OR, 1.11; 95% CI, 1.01-1.23) vs 18 to 25 years, were divorced (OR, 1.12; 95% CI, 1.04-1.21) or separated (OR, 1.19; 95% CI, 1.07-1.32) vs single or never married, were former (OR, 1.18; 95% CI, 1.10-1.28) or current smokers (OR, 1.67; 95% CI, 1.58-1.78) vs nonsmokers, and were former (OR, 1.26; 95% CI, 1.16-1.37) or current drinkers (OR, 1.37; 95% CI, 1.29-1.47) vs never drinkers. Less than high school (OR, 1.75; 95% CI, 1.59-1.93), high school graduate (OR, 1.45; 95% CI, 1.32-1.59), or some college or associate’s degree (OR, 1.40; 95% CI, 1.28-1.54) was also associated with psychological distress compared with a college degree or higher.

**Table 2.** Multivariable Logistic Regression Analysis of Psychological Distress and Associated Risk Factors Among US-Born vs Non–US-Born Black or African American Adults

Variable	OR (95% CI)^a^					
	Overall		Non–US-born		US-born	
	Unadjusted	Adjusted	Unadjusted	Adjusted	Unadjusted	Adjusted
Age, y						
18-25	1 [Reference]	1 [Reference]	1 [Reference]	1 [Reference]	1 [Reference]	1 [Reference]
26-34	0.99 (0.90-1.09)	1.10 (1.01-1.21)^b^	0.83 (0.61-1.12)	1.03 (0.74-1.42)	1.03 (0.93-1.13)	1.11 (1.01-1.23)^b^
35-44	0.91 (0.83-1.00)	1.03 (0.93-1.14)	0.89 (0.67-1.18)	1.08 (0.79-1.47)	0.93 (0.84-1.03)	1.02 (0.92-1.14)
45-54	1.09 (0.99-1.19)	1.11 (1.01-1.23)^b^	0.81 (0.59-1.11)	0.96 (0.68-1.37)	1.14 (1.04-1.25)^c^	1.13 (1.02-1.25)^b^
55-64	1.08 (0.98-1.19)	0.94 (0.84-1.04)	0.79 (0.58-1.06)	0.80 (0.57-1.12)	1.11 (1.01-1.23)^b^	0.94 (0.84-1.06)
≥65	0.71 (0.64-0.78)^d^	0.51 (0.44-0.58)^d^	0.85 (0.61-1.18)	0.64 (0.43-0.94)^b^	0.70 (0.63-0.77)^d^	0.51 (0.44-0.58)^d^
Sex						
Male	0.71 (0.67-0.75)^d^	0.68 (0.56-0.82)^d^	0.63 (0.53-0.74)^d^	0.68 (0.56-0.82)^d^	0.73 (0.69-0.77)^d^	0.71 (0.66-0.75)^d^
Female	1 [Reference]	1 [Reference]	1 [Reference]	1 [Reference]	1 [Reference]	1 [Reference]
Marital status						
Divorced	0.98 (0.92-1.05)	1.12 (1.04-1.21)^c^	1.04 (0.82-1.32)	1.06 (0.83-1.37)	0.98 (0.91-1.06)	1.12 (1.04-1.22)^c^
Widowed	0.87 (0.79-0.95)^c^	1.05 (0.94-1.17)	1.16 (0.82-1.63)	1.05 (0.71-1.55)	0.85 (0.77-0.93)^c^	1.06 (0.94-1.19)
Separated	1.25 (1.13-1.38)^d^	1.19 (1.07-1.32)^c^	1.00 (0.76-1.31)	0.91 (0.68-1.23)	1.31 (1.18-1.46)^d^	1.23 (1.10-1.37)^d^
Married or living with a partner	0.69 (0.65-0.73)^d^	0.91 (0.85-0.97)^c^	0.57 (0.48-0.69)^d^	0.63 (0.52-0.78)^d^	0.72 (0.68-0.77)^d^	0.95 (0.89-1.02)
Single or never married	1 [Reference]	1 [Reference]	1 [Reference]	1 [Reference]	1 [Reference]	1 [Reference]
Region of residence						
Northeast	1 [Reference]	1 [Reference]	1 [Reference]	1 [Reference]	1 [Reference]	1 [Reference]
North Central or Midwest	1.18 1.07-1.31)^c^	1.09 (0.99-1.20)	0.92 (0.69-1.22)	0.99 (0.74-1.32)	1.15 (1.04-1.29)^c^	1.11 (0.99-1.24)
South	1.06 (0.97-1.15)	1.04 (0.96-1.13)	0.85 (0.69-1.04)	0.95 (0.76-1.18)	1.04 (0.95-1.14)	1.06 (0.97-1.16)
West	1.13 (1.00-1.27)	1.17 (1.04-1.31)^c^	1.08 (0.79-1.49)	1.17 (0.84-1.63)	1.10 (0.97-1.26)	1.18 (1.04-1.34)^b^
Employment status						
Employed	1 [Reference]	1 [Reference]	1 [Reference]	1 [Reference]	1 [Reference]	1 [Reference]
Not employed	1.99 (1.89-2.09)^d^	1.91 (1.80-2.03)^d^	1.71 (1.45-2.01)^d^	1.50 (1.23-1.82)^d^	1.99 (1.89-2.11)^d^	1.97 (1.84-2.09)^d^
Health insurance status						
Insured	0.74 (0.70-0.79)^d^	0.91 (0.85-0.98)^c^	0.84 (0.69-1.01)	0.95 (0.76-1.19)	0.72 (0.67-0.77)^d^	0.90 (0.83-0.97)^c^
Uninsured	1 [Reference]	1 [Reference]	1 [Reference]	1 [Reference]	1 [Reference]	1 [Reference]
Education						
Less than high school	2.69 (2.46-2.94)^d^	1.75 (1.59-1.93)^d^	1.88 (1.46-2.41)^d^	1.28 (0.97-1.68)	2.78 (2.52-3.06)^d^	1.82 (1.64-2.03)^d^
High school graduate	1.94 (1.78-2.11)^d^	1.45 (1.32-1.59)^d^	1.65 (1.31-2.06)^d^	1.35 (1.06-1.71)^b^	1.96 (1.78-2.15)^d^	1.46 (1.32-1.62)^d^
Some college or associate’s degree	1.80 (1.65-1.97)^d^	1.40 (1.28-1.54)^d^	1.58 (1.26-1.99)^d^	1.29 (1.02-1.63)^b^	1.81 (1.65-2.00)^d^	1.42 (1.29-1.57)^d^
College degree or higher	1 [Reference]	1 [Reference]	1 [Reference]	1 [Reference]	1 [Reference]	1 [Reference]
Poverty status						
Income less than poverty threshold	1 [Reference]	1 [Reference]	1 [Reference]	1 [Reference]	1 [Reference]	1 [Reference]
Income greater than or equal to poverty threshold	0.44 (0.42-0.47)^d^	0.67 (0.64-0.72)^d^	0.52 (0.45-0.62)^d^	0.73 (0.60-0.88)^c^	0.44 (0.41-0.46)^d^	0.67 (0.63-0.72)^d^
Body mass index^e^						
Underweight or normal (<25)	1 [Reference]	1 [Reference]	1 [Reference]	1 [Reference]	1 [Reference]	1 [Reference]
Overweight (≥25 to <30)	0.88 (0.82-0.94)^d^	0.98 (0.92-1.05)	0.94 (0.77-1.14)	1.08 (0.89-1.31)	0.87 (0.81-0.93)^d^	0.97 (0.90-1.04)
Obesity (≥30)	1.21 (1.14-1.29)^d^	1.24 (1.16-1.32)^d^	1.57 (1.30-1.90)^d^	1.58 (1.29-1.93)^d^	1.16 (1.09-1.23)^d^	1.20 (1.13-1.29)^d^
Physical activity						
Inactive or insufficient	1 [Reference]	1 [Reference]	1 [Reference]	1 [Reference]	1 [Reference]	1 [Reference]
Physically active	1.02 (0.86-1.21)	1.05 (0.89-1.25)	1.07 (0.63-1.81)	1.15 (0.68-1.94)	1.01 (0.84-1.21)	1.05 (0.87-1.26)
Alcohol drinking status						
Never	1 [Reference]	1 [Reference]	1 [Reference]	1 [Reference]	1 [Reference]	1 [Reference]
Former	1.30 (1.20-1.40)^d^	1.26 (1.16-1.37)^d^	1.26 (0.98-1.60)	1.14 (0.88-1.48)	1.29 (1.19-1.39)^d^	1.29 (1.19-1.41)^d^
Current	1.28 (1.20-1.36)^d^	1.37 (1.29-1.47)^d^	0.85 (0.72-1.00)	0.88 (0.73-1.07)	1.33 (1.24-1.42)^d^	1.45 (1.36-1.56)^d^
Smoking status						
Never	1 [Reference]	1 [Reference]	1 [Reference]	1 [Reference]	1 [Reference]	1 [Reference]
Former	1.20 (1.12-1.29)^d^	1.18 (1.10-1.28)^d^	1.28 (1.00-1.65)	1.56 (1.18-2.05)^c^	1.18 (1.10-1.26)^d^	1.16 (1.07-1.25)^d^
Current	2.15 (2.03-2.28)^d^	1.67 (1.58-1.78)^d^	1.85 (1.45-2.36)^d^	1.98 (1.52-2.59)^d^	2.12 (2.00-2.26)^d^	1.63 (1.53-1.73)^d^

Abbreviation: OR, odds ratio.

^a^ Unadjusted ORs include 1 variable. Adjusted ORs are adjusted for all variables.

^b^ Indicates statistical significance at *P* < .05.

^c^ Indicates statistical significance at *P* < .01.

^d^ Indicates statistical significance at *P* < .001.

^e^ Body mass index is calculated as weight in kilograms divided by height in meters squared.

**Figure 2.**
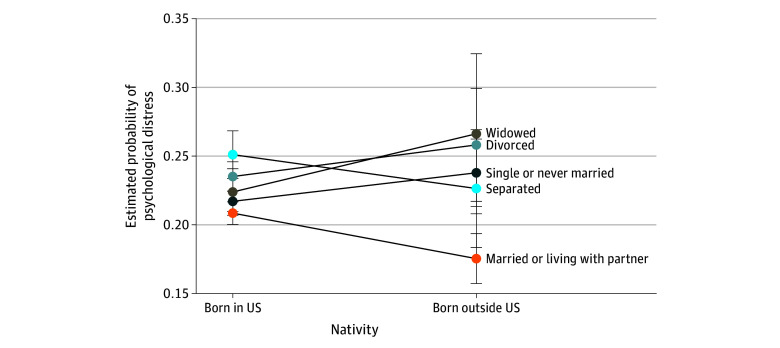
Estimated Probability of Psychological Distress by Nativity and Marital Status Intersections

**Figure 3.**
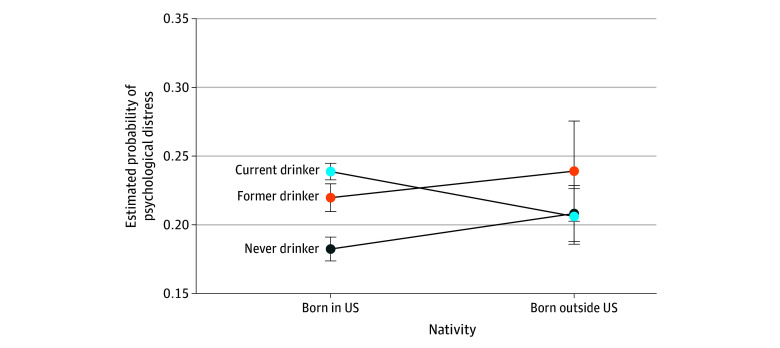
Estimated Probability of Psychological Distress by Nativity and Alcohol Drinking Status Intersections

Significant interactions were found only between nativity and marital status (*F*_4,1193_ = 4.58; *P* = .001) and nativity and alcohol drinking status (*F*_2,1195_ = 8.70; *P* < .001), adjusting for the remaining factors. The interaction effects of nativity and marital status showed that non–US-born individuals who were married or living with a partner had the lowest probability of experiencing psychological distress (Figure 2). Similarly, for the interaction effects of nativity and alcohol drinking status, US-born individuals who had never consumed alcohol had the lowest of experiencing psychological distress (Figure 3).

Among US-born individuals, lower odds were observed for those who were insured (OR, 0.90; 95% CI, 0.83-0.97) vs uninsured. Higher odds were observed for those who were aged 26 to 34 years (OR, 1.11; 95% CI, 1.01-1.23) or 45 to 54 years (OR, 1.13; 95% CI, 1.02-1.25) vs 18 to 25 years, were divorced (OR, 1.12; 95% CI, 1.04-1.22) or separated (OR, 1.23; 95% CI, 1.10-1.37) vs single or never married, were living in the West (OR, 1.18; 95% CI, 1.04-1.34) vs Northeast, and were former (OR, 1.29; 95% CI, 1.19-1.41) or current alcohol drinkers (OR, 1.45; 95% CI, 1.36-1.56) vs never drinkers (Table 2).

Within the non–US-born subgroup, being married or living with a partner was associated with lower odds of psychological distress (OR, 0.63; 95% CI, 0.52-0.78) compared with being single or never married. Region of residence, health insurance, and alcohol drinking were not significantly associated with psychological distress (Table 2). Length of stay in the US was also not associated with psychological distress in this group (eTable 3 in Supplement 1).

## Discussion

To our knowledge, this cross-sectional study is the first population-based study disaggregating data to explore associations of sociodemographic, socioeconomic, and health behavior factors with moderate-severe psychological distress in the overall Black or African American adult population and their US-born and non–US-born subgroups. Some studies^4,5,38,39,40^ have indicated that immigrants and Black or African American individuals experience more psychological distress than US-born and White individuals, respectively. However, previous studies often examine Black or African Americans as a monolithic group, potentially obscuring critical nuances in between-group and within-group differences. This study aimed to address these gaps by providing a more comprehensive understanding of psychological distress in these populations.

Contrary to existing research arguing that immigrants experience higher levels of psychological distress than their US-born counterparts,^39,40^ our results found that the US-born subgroup experienced greater psychological distress than the non–US-born subgroup. This may be related to differing internalizations of discriminatory experiences based on nativity. Discrimination has been linked to exacerbated stress and mental health issues, with US-born individuals reporting substantially more discrimination than non–US-born individuals.^41,42,43,44^ Therefore, minoritized status, coupled with a more-acute awareness of discriminatory experiences, may put US-born Black or African American adults at greater risk of psychological distress. However, although non–US-born Black adults may be less affected by discrimination initially, longer exposure to the host society can lead to increased ethnic consciousness and perceptiveness to microaggressions or prejudiced behaviors.^44^ More research is needed to explore how acculturation impacts psychological distress and mental health outcomes among non–US-born populations.

Our findings showed marked variations in the associations of moderate-severe psychological distress with sociodemographic and socioeconomic factors. Consistent with previous studies,^45,46^ disadvantaged groups, including female individuals, unemployed persons, and those with incomes below the poverty threshold, were more likely to experience psychological distress, regardless of nativity. Individuals who had obesity, were current smokers, and had less than a high school education also reported a higher prevalence of moderate-severe psychological distress. These findings may be indicative of how certain characteristics shared by disadvantaged populations increase the risk of psychological distress regardless of nativity. Recent literature^47,48^ suggests that behavioral health problems and lower quality of life negatively interfere with cognitive control over certain neurotransmitters (eg, dopamine, serotonin, norepinephrine, and glutamate) such that risk of psychological distress becomes elevated. This implies that among disadvantaged communities, such as Black or African American adults, increased experiences of psychological distress could be related to aspects of behavioral health or socioeconomic status that lead to daily, cumulative, or chronic stress (eg, poor housing, overcrowding, food insecurity, and lack of health resources and transportation).^48^

It should be noted that poverty, obesity, and smoking were more potent factors among non–US-born individuals. Unemployment and education were more notable factors among US-born individuals. Our finding regarding smoking status contributes to the current literature, which primarily examines a unidirectional relationship in which psychological distress influences smoking behaviors, rather than considering bidirectional effects.^49,50^ This highlights the need to investigate how such health behaviors may serve as risk factors rather than solely as outcomes of mental health conditions. Regarding BMI, the increased risk among non–US-born individuals may partly result from the replacement of traditional dietary and physical activity habits with less healthy behaviors prevalent in host countries.^51^ Furthermore, poverty as a factor for non–US-born individuals associated with financial obligations, such as remittances, and the stresses of maintaining kinship and household well-being in host and home countries.^52^

On the other hand, unemployment and education as factors among US-born individuals may be associated with systemic racism and the diminished return of educational attainment on Black adults’ subjective health.^53^ Although higher educational attainment has been associated with better mental health among US adults,^54^ research suggests that this might not apply to immigrants, who report better health despite often having lower educational attainment.^55^ Past research also suggests that non–US-born individuals may be more likely than their US-born counterparts to accept less desirable jobs with inadequate insurance or benefits, which may reduce the relative harm of unemployment.^56^

Marital status yielded nuanced results. The significant interaction between nativity and marital status revealed that being a non–US-born individual who was married or living with a partner was associated with the lowest probability of experiencing psychological distress vs other marital statuses irrespective of nativity. Since social support is associated with decreased mental health symptoms,^57^ being married or living with a partner may be a source of considerable social support for non–US-born individuals. Stratification by nativity also revealed differences regarding alcohol drinking status. Past research has associated alcohol drinking with higher depressive symptoms and lower well-being among Black drinkers.^58^ However, when stratified by nativity, we found that alcohol drinking was not associated with higher odds of psychological distress among non–US-born individuals. Alcohol consumption is higher in North American countries than in African countries and is significantly associated with increased risk of psychological distress.^59,60,61^ Subsequently, increased socialization and exposure to American drinking culture may place US-born Black or African American adults at higher risk for hazardous alcohol consumption, which could, in turn, contribute to greater psychological distress.^62^ Understanding these dynamics will be crucial in developing targeted interventions to support mental well-being in these communities.

Having health insurance was associated with lower odds of psychological distress within the US-born subgroup, but not the non–US-born subgroup. This may be due to immigrants’ underutilization of the health care system. Previous studies^63,64^ have found that the prevalence of mental health problems among Black immigrants does not correlate with mental health treatment-seeking behaviors, as they are less likely to utilize mental health services despite experiencing mental health issues. Reasons for underutilization may include mental health stigmatization, distrust in the health care system, and a tendency to seek comfort from other sources, such as religiosity and social supports.^64,65,66,67,68^ Subsequently, our findings underscore the need to evaluate the US health care system, which stands to benefit from increasing access and adaptability in mental health treatments.

### Limitations

This study had some limitations. First, causal inferences cannot be made owing to the cross-sectional nature of NHIS data. Second, because the data are based on self-reported survey responses, they may have been impacted by respondents’ social desirability biases. Third, although many factors were considered in our analyses, residual confounding factors (eg, English proficiency or citizenship) may have further influenced the observed associations. Future research should consider the inclusion of language, acculturation, and/or citizenship status as discrete factors for health outcomes.

## Conclusions

Our findings highlight important within-group and between-group differences in the prevalence of and factors associated with moderate-severe psychological distress among Black or African American adults in the US. Overall, this study found that, contrary to existing assumptions, US-born Black or African American adults demonstrated higher prevalence and risks of moderate-severe psychological distress compared with their non–US-born counterparts. By disaggregating data, this study found shared and nuanced associations in behavioral and other health-related factors of psychological distress by nativity. Subsequently, data disaggregation becomes particularly relevant for communities such as Black or African American populations, where immigrant subgroups are rapidly growing, and where ethnic identity, assimilation, and acculturation can impact health differently depending on background.^17,69,70^

## Notes

Supplement 1. **eTable 1.** The Unadjusted and Adjusted Odds of Psychological Distress Associated With Nativity Status Among Black or African American Adults (n= 49,820) **eTable 2.** The Prevalence and Odds of Psychological Distress Associated With Years in the US Among Black or African American Adults (n= 49,820) **eTable 3.** The Prevalence and Odds of Psychological Distress Associated With Years in the US Among Black Non–US-Born Adults (n= 5,935)

Supplement 2.
Data Sharing Statement
