# Supplementary material for: Psychological Distress Among US-Born and Non–US-Born Black or African American Adults in the US
Source: JAMA Netw Open. 2025 Apr 28;8(4):e256558. doi: 10.1001/jamanetworkopen.2025.6558 (PMC12038514; doi:10.1001/jamanetworkopen.2025.6558)
Supplement: Supplement 1. — eTable 1. The Unadjusted and Adjusted Odds of Psychological Distress Associated With Nativity Status Among Black or African American Adults (n= 49,820) eTable 2. The Prevalence and Odds of Psychological Distress Associated With Years in the US Among Black or African American Adults (n= 49,820) eTable 3. The Prevalence and Odds of Psychological Distress Associated With Years in the US Among Black Non–US-Born Adults (n= 5,935) [file jamanetwopen-e256558-s001.pdf]

Supplemental Online Content

Elhabashy M, Adzrago D, Williams F. Psychological distress among US-born and non–US-born Black or African American adults in the US. *JAMA Netw Open*. 2025;8(4):e256558. doi:10.1001/jamanetworkopen.2025.6558

eTable 1. The Unadjusted and Adjusted Odds of Psychological Distress Associated With Nativity Status Among Black or African American Adults (n= 49,820)

eTable 2. The Prevalence and Odds of Psychological Distress Associated With Years in the US Among Black or African American Adults (n= 49,820)

eTable 3. The Prevalence and Odds of Psychological Distress Associated With Years in the US Among Black Non–US-Born Adults (n= 5,935)

This supplemental material has been provided by the authors to give readers additional information about their work.

© 2025 Elhabashy M et al. *JAMA Network Open*.

eTable 1. The Unadjusted and Adjusted Odds of Psychological Distress Associated With Nativity Status Among Black or African American Adults (n= 49,820)

	Total sample<br>N (% [95% CI])	Crude or unadjusted OR (95% CI)	Adjusted OR (95% CI)
**Nativity**			
Black US-born	43,885 (88.1 [87.5, 88.7])	1 [Reference]	1 [Reference]
Black non–US-born	5,935 (11.9 [11.3, 12.5])	0.72*** (0.66, 0.80)	0.72*** (0.65, 0.79)

Frequencies are unweighted while percentages are weighted.

*OR = odds ratio. 95% CI = 95% confidence interval. Statistical significance at *p<0.05, **p<0.01, and ***p<0.001.*

Adjusted OR: adjusted for age.

eTable 2. The Prevalence and Odds of Psychological Distress Associated With Years in the US Among Black or African American Adults (n= 49,820)

	Total sample<br>N (% [95% CI])	Psychological distress (Yes)<br>n (% [95% CI])	p-value	Crude or unadjusted<br>OR (95% CI)	Adjusted OR (95% CI)
**Length of stay in the US**					
<5 years	622 (1.3 [1.2, 1.5])	113 (17.8 [14.7, 21.4])	**<0.001**	0.74* (0.59, 0.94)	0.73** (0.58, 0.92)
5 to <10 years	846 (1.7 [1.6, 1.9])	148 (16.8 [14.1, 19.8])		0.69*** (0.56, 0.85)	0.68*** (0.55, 0.83)
10 to <15 years	877 (1.7 [1.6, 1.9])	130 (15.5 [12.9, 18.5])		0.63*** (0.51, 0.78)	0.62*** (0.50, 0.77)
15 years or more	3,590 (7.1 [6.7, 7.6])	651 (18.0 [16.3, 19.7])		0.75*** (0.67, 0.85)	0.75*** (0.66, 0.84)
US-born	43,885 (88.1 [87.5, 88.7])	10,037 (22.6 [22.0, 23.1])		1 [Reference]	1 [Reference]

Frequencies are unweighted while percentages are weighted.

P-value for the prevalence was based on Rao-Scott chi-square test, a design-adjusted version of the Pearson chi-square test to determine bivariate differences in the psychological distress prevalence by nativity status among Black or African American adults.

*OR = odds ratio. 95% CI = 95% confidence interval. Statistical significance at *p<0.05, **p<0.01, and ***p<0.001.*

Adjusted OR: adjusted for age.

© 2025 Elhabashy M et al. *JAMA Network Open*.

eTable 3. The Prevalence and Odds of Psychological Distress Associated With Years in the US Among Black Non–US-Born Adults (n= 5,935)

	All non–US-born sample N (% [95% CI])	Psychological distress (Yes) n (% [95% CI])	p-value	Crude or unadjusted OR (95% CI)	Adjusted OR (95% CI)
**Length of stay in the US**					
<5 years	622 (11.2 [10.2, 12.4])	113 (17.8 [14.7, 21.4])	0.49	1 [Reference]	1 [Reference]
5 to <10 years	846 (14.5 [13.4, 15.6])	148 (16.8 [14.1, 19.8])		0.93 (0.68, 1.27)	0.94 (0.69, 1.30)
10 to <15 years	877 (14.3 [13.3, 15.4])	130 (15.5 [12.9, 18.5])		0.85 (0.63, 1.13)	0.88 (0.66, 1.19)
15 years or more	3,590 (60.0 [58.2, 61.7])	651 (18.0 [16.3, 19.7])		1.01 (0.78, 1.31)	1.11 (0.83, 1.47)
US-born	N/A	N/A		N/A	N/A

Frequencies are unweighted while percentages are weighted.

P-value for the prevalence was based on Rao-Scott chi-square test, a design-adjusted version of the Pearson chi-square test to determine bivariate differences in the psychological distress prevalence by nativity status among Black or African American adults.

*OR = odds ratio. 95% CI = 95% confidence interval. Statistical significance at *p<0.05, **p<0.01, and ***p<0.001.*

Adjusted OR: adjusted for age.

© 2025 Elhabashy M et al. *JAMA Network Open*.
