# Supplementary material for: Psychological Distress Among US-Born and Non–US-Born Black or African American Adults in the US
Source: JAMA Netw Open. 2025 Apr 28;8(4):e256558. doi: 10.1001/jamanetworkopen.2025.6558 (PMC12038514; doi:10.1001/jamanetworkopen.2025.6558)
Supplement: Supplement 2. — Data Sharing Statement [file jamanetwopen-e256558-s002.pdf]

Data Sharing Statement

Elhabashy. Psychological Distress Among US-Born and Non–US-Born Black or African American Adults in the US. *JAMA Netw Open*. Published April 28, 2025. doi:10.1001/jamanetworkopen.2025.6558

Data
Data available: No

Additional Information
Explanation for why data not available: The datasets generated for the study are publicly available in the CDC database repository at https://www.cdc.gov/nchs/nhis/data-questionnaires-documentation.htm.
